# Advanced Pseudocapacitive Performances of a Ti_3_C_2_T_x_–ZnOHF/ZnO Nanocomposite for Energy Storage Applications

**DOI:** 10.1002/cssc.202500024

**Published:** 2025-07-08

**Authors:** Gisella M. Di Mari, Chengning Yao, Tianhao Lan, Sihui Liu, Giacometta Mineo, Vincenzina Strano, Elena Bruno, Ji‐Seon Kim, Salvatore Mirabella, Felice Torrisi

**Affiliations:** ^1^ Department of Physics and Astronomy University of Catania,“Ettore Majorana” via S. Sofia 64 95123 Catania Italy; ^2^ Consiglio Nazionale per le Ricerche, Istituto per la MIcroelettronica e i Microsistemi (CNR‐IMM) Catania Università via S. Sofia 64 95123 Catania Italy; ^3^ Molecular Sciences Research Hub, Department of Chemistry & Centre for Processable Electronics Imperial College London White City Campus, 82 Wood Lane London W12 0BZ UK; ^4^ Department of Physics and Centre for Processable Electronics Imperial College London London SW7 2AZ UK

**Keywords:** 2D materials, energy storage, high‐performance capacitors, MXenes, neutral pH, supercapacitors, zinc oxide nanostars

## Abstract

The growing demand for efficient and high‐performance energy storage systems is driving the exploration of novel materials and composites. Traditional electrode materials often face limitations in terms of energy and power densities. This article demonstrates a novel spray‐coated cathode electrode system composed of Ti_3_C_2_T_*x*_ MXene and zinc hydroxy fluoride/zinc oxide nanostars for energy storage applications in a neutral pH electrolyte (1M Na_2_SO_4_), thus avoiding corrosion problems related to water splitting reactions. Optimized Ti_3_C_2_T_*x*_‐nanostar electrodes exhibit superior specific capacitance, achieving 236 F g^−1^ at 5 mV s^−1^ in cyclic voltammetry and 139 F g^−1^ at 5 mV s^−1^ in galvanostatic charge–discharge measurements, which is superior to pure Ti_3_C_2_T_*x*_ (115 F g^−1^ at 0.5 A g^−1^) and pure nanostar (108 F g^−1^ at 0.5 F g^−1^) electrodes, used as reference. Additionally, an asymmetric Ti_3_C_2_T_*x*_||Ti_3_C_2_T_*x*_‐nanostars supercapacitor device achieves a specific capacitance of 147 F g^−1^ at 0.5 A g^−1^, an energy density *E*_d_ ≈ 46 W h kg^−1^ at a power density *P*_d_ ≈ 875 W kg^−1^, and the highest *P*_d_ ≈ 16 650 W kg^−1^ at *E*_d_ ≈ 14 W h kg^−1^. These findings demonstrate that zinc oxide nanostars combined with delaminated Ti_3_C_2_T_*x*_ MXene hold a significant promise for efficient energy storage applications, leveraging the synergy between double‐layer capacitance and pseudocapacitive effects.

## Graphical abstract

MXene and zinc‐based nanostructures are investigated as hybrid electrodes for energy storage. The hybrid electrodes are prepared via spray coating and evaluated as cathode materials for supercapacitors in neutral electrolytes. We find an enhancement of the specific capacitance to 236 F g^−1^ at 5 mV s^−1^. Our supercapacitors using the hybrid cathode demonstrate higher energy and power densities over other relevant studies.

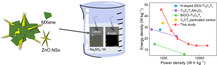

## 1 Introduction

The environmental impact and the decreasing availability of fossil fuels are primarily recognized as a leading causes of climate change, driving an increasing demand for renewable energy sources (i.e., solar and wind energy^[^ ^1^ ^]^) to facilitate sustainable development. Renewable energy sources are inherently intermittent; hence their widespread use requires the development of energy storage devices capable of efficiently storing and delivering energy whenever there is a need.^[^ ^2^ ^]^

The development of novel and high‐performance energy storage devices entails a shift towards more sustainable and environmentally‐friendly materials.^[^ ^3^ ^]^

Lithium‐ion batteries are widely utilized in energy storage, including variants like lithium‐cobalt‐oxide (LCO), nickel‐cobalt‐manganese (NCM), and lithium‐iron‐phosphate (LFP).^[^ ^4^ ^]^ Batteries typically exhibit higher energy densities and lower power densities, compared to supercapacitors. Supercapacitors offer numerous advantages, including high‐power density (103–104 W kg^−1^), quicker charge and discharge rates (from seconds to minutes, depending on the applied voltage), and current and extended lifespan (they can last for 500 000 to 1 000 000 cycles or more without significant degradation).^[^ ^5^ ^]^ The choice between batteries and supercapacitors largely depends on the specific demands of the application. Additionally, a third category of devices, known as pseudocapacitors, exists, which strikes a balance between the benefits of batteries and supercapacitors, maintaining both high energy and power densities.^[^ ^6^ ^]^ The typical pseudocapacitive materials are transition metal oxides, which exhibit various oxidation states, enabling them to undergo diverse redox reactions.^[^ ^6^ ^]^ In storage research, aqueous electrolytes are commonly classified into three categories based on pH: acidic (e.g., H_2_SO_4_), alkaline (e.g., KOH, NaOH), and neutral (e.g., Na_2_SO_4_, KCl). While acidic and alkaline media can promote faster kinetics, they typically compromise material stability due to corrosion and structural deterioration. Neutral electrolytes, by minimizing these effects, ensure greater durability and safety, which are critical for real‐world applications.^[^ ^7^ ^]^

Among transition metal oxides, Zinc Oxide (ZnO) is a promising material for pseudocapacitors, thanks to its low cost, abundancy, and morphology variation.^[^ ^8^, ^9^ ^]^ ZnO is a pseudocapacitive material capable of reaching high‐specific capacitances (≈150 F g^−1^ at 5 mV s^−1^ in neutral pH^[^ ^10^, ^11^ ^]^); when coupled with other materials (i.e., carbon‐based materials^[^ ^10^ ^]^), ZnO enables electrodes with specific capacitances ranging from 200 to 300 F g^−1^ at 5 mV s^−1^ in neutral pH condition.

Combining ZnO with other materials, such as carbon‐based materials, can contribute to further enhancing the ZnO mechanical stability, enhance electrical conductivity or increase energy storage capacity.^[^ ^12^, ^13^, ^14^ ^]^ For example, Guo et al.^[^ ^15^ ^]^ prepared a ZnO/Graphene composite through microwave irradiation for energy storage, obtaining a specific capacitance of 201 F g^−1^ at 1 A g^−1^ in 1 M Na_2_SO_4_, far beyond the bare materials (77 F g^−1^ for MXene, 5 F g^−1^ for bare ZnO).

MXenes, an emerging class of advanced 2D materials,^[^ ^16^ ^]^ are gaining interest thanks to their outstanding performance in energy storage applications^[^ ^17^ ^]^ and wearable electronics.^[^ ^18^ ^]^ Their high‐surface area,^[^ ^19^ ^]^ and the unique 2D structure, allow for an enhanced interaction with electrolytes in energy storage devices, such as batteries and supercapacitors.^[^ ^20^ ^]^ Ali et al.^[^ ^21^ ^]^ tested a hybrid NiMn‐metal organic framework (MOF)/MXene device, in alkaline media, revealing a capacity of 215.5 F g^−1^, an energy density of 74.4 W h kg^−1^, and a power output of 1600 W kg^−1^ at a current density of 1.0 A g^−1^. The device demonstrated 94% coulombic efficiency and 90% capacity retention over 5000 galvanostatic charge–discharge (GCD) cycles. These impressive results are attributed to the electrochemical properties of MXene nanosheets, whose 2D structure and abundant surface functional groups provide a high‐surface area. When combined with NiMn MOF, MXene enhances the overall surface area, increasing active sites for ion adsorption/desorption, thereby boosting charge storage and electrode kinetics.

Ti_3_C_2_T_*x*_ MXene‐ZnO nanocomposites have demonstrated excellent performances in fields, such as Hydrogen production, photocatalysis, and gas sensing. Liu et al.^[^ ^22^ ^]^ prepared Ti_3_C_2_T_*x*_ MXene sheets, decorated by ZnO nanorods for pollutants removal in water by photocatalysis. Such MXene‐ZnO hybrids showed a removal ability of Rhodamine B of about 3.2 times faster than pure ZnO nanorods. Zhu et al.^[^ ^23^ ^]^ prepared a ZnO‐based gas sensor, containing different proportions of Ti_3_C_2_T_*x*_, synthesized by hydrothermal method. They evaluated gas‐sensing properties of ZnO:Ti_3_C_2_T_*x*_ composites at different proportions of MXene content. The ZnO:Ti_3_C_2_T_*x*_ composites containing 2 wt% Ti_3_C_2_T_*x*_ had the highest response to 14.4–100 ppm of acetone. Similarly, MXene could contribute to enhancing the ZnO conductivity in hybrid electrodes, leading to higher performances in energy storage applications. We previously reported a unique zinc hydroxy fluoride ZnOHF‐ZnO based structure, called Nanostar (NS), made of bundles of crystalline ZnO nanostrips (30 nm thin and up to 12 μm long) with a six‐point star shape, self‐assembled onto a plane. The structure achieved excellent storage performances of ≈100 F g^−1^ in 1 M Na_2_SO_4_. MXene‐enhanced ZnO NSs could combine the advantages of MXenes and ZnO nanostructures, unleashing their potential for energy storage applications. In this work, we demonstrate a Ti_3_C_2_T_*x*_ MXene/ZnOHF‐ZnO NSs (Ti_3_C_2_T_*x*_‐NSs) nanocomposite as a viable material to produce cathodic electrodes for pseudocapacitors in energy storage applications. A complete characterization of the material was performed to confirm the sample morphology, composition, and crystal structure through scanning electron microscopy (SEM), atomic force microscopy (AFM), X‐ray diffraction spectroscopy (XRD), Raman spectroscopy, and X‐Ray photoelectron spectroscopy (XPS). The electrochemical characterization of Ti_3_C_2_T_*x*_‐NSs composites revealed improved energy storage performances with respect to that of isolated Ti_3_C_2_T_*x*_ or NSs materials. Different Ti_3_C_2_T_*x*_‐NSs ratios in the composite were investigated in order to optimize it as a cathode for pseudocapacitors with improved energy‐storage performances. A cathode with a 1:5 Ti_3_C_2_T_*x*_‐NSs ratio results in a pseudocapacitor with a specific capacitance of 236 F g^−1^ at 5 mV s^−1^, which is almost doubled respect to the with Ti_3_C_2_T_*x*_ electrodes (140 F g^−1^ at 5 mV s^−1^). Finally, the electrochemical performance of the Ti_3_C_2_T_*x*_‐NSs 1:5 cathode was investigated in an asymmetric device of Ti_3_C_2_T_*x*_ || Ti_3_C_2_T_*x*_‐NSs 1:5 and compared to that of a symmetric Ti_3_C_2_T_*x*_ || Ti_3_C_2_T_*x*_ device. The extrapolated energy density (*E*_d_) and power density (*P*_d_) of our Ti_3_C_2_T_*x*_‐NSs device are superior to other MXene‐based devices working in neutral electrolytes, reaching *E*_d_ ≈ 46 W h kg^−1^ at a *P*_d_ ≈ 875 W kg^−1^ and a *P*_d_ ≈ 16 650 W kg^−1^ at an *E*_d_ ≈ 14 W h kg^−1^. The Ti_3_C_2_T_*x*_‐NSs composite material shows promising potential for future applications as a supercapacitor, offering exceptional performance that could enhance energy storage systems in advanced electronic devices and sustainable energy solutions.

## 2 Results and Discussion

**Figure 1:**
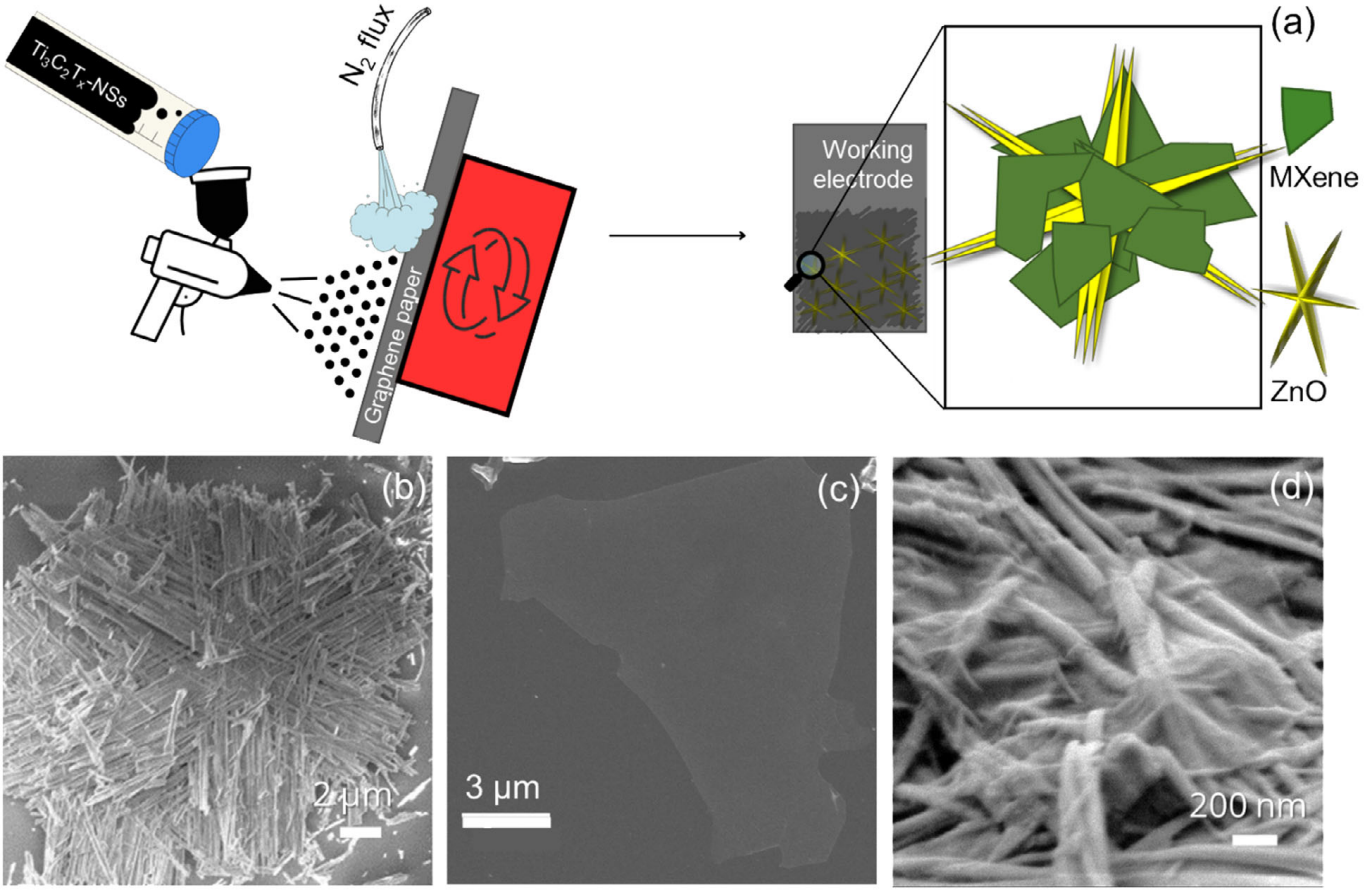
a) Schematic of sample preparation for electrochemical characterization, working electrode schematization, and 3D rendering of the Ti_3_C_2_T_*x*_‐NSs nanocomposite; b) NSs SEM image; c) Ti_3_C_2_T_*x*_ SEM image; d) medium (tilted view) magnification of the Ti_3_C_2_T_*x*_‐NSs 1:5 electrode.

**Figure** **1**a reports a schematic of the electrodes' preparation (see the experimental section for further details). A Ti_3_C_2_T_*x*_‐NSs aqueous solution is spray coated onto graphene paper (GP, see methods). The obtained working electrode (WE), (centre in Figure 1a), is then characterized in a three‐electrode system. A 3D rendering of Ti_3_C_2_T_*x*_‐NSs structure is represented in right in Figure 1a. Figure 1b shows an SEM micrograph of a typical NS structure. The NSs are made of six arms, sharing a common center. These arms are made of groups of parallel wires. The nanostructure can be circumscribed in a circle with a radius of 5 μm, and the arms are on the plane at a 60° angle to each other. AFM statistics for Ti_3_C_2_T_*x*_ are reported in Figure S1a,b, Supporting Information, presents a high‐resolution AFM micrograph of a monolayer Ti_3_C_2_T_*x*_ flake, with the height profile over the lateral length reported in figure S1(c), Supporting Information, Figure 1c shows an SEM micrograph of a typical Ti_3_C_2_T_*x*_ flake. Figure 1d shows a medium magnification SEM image of a Ti_3_C_2_T_*x*_‐NSs (1:5 weight ratio) spray‐coated electrode (tilted view). The Ti_3_C_2_T_*x*_ flakes are present on the NSs, and conform to the NSs morphology, confirming that both materials are physically in intimate contact with each other. Noteworthy, the SEM image shown in Figure 1d is acquired after the sample electrochemical characterization, indicating that the electrochemical characterization did not destroy the electrode structure.

**Figure 2:**
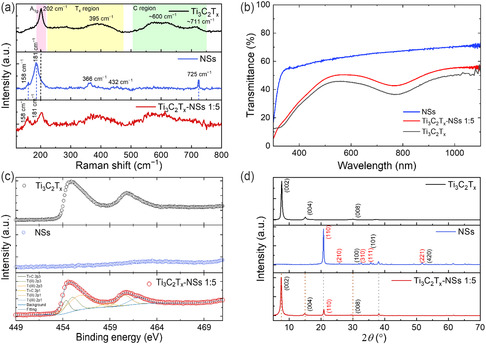
a) Raman Spectra of Ti_3_C_2_T_*x*_ (black), NSs (blue) and Ti_3_C_2_T_*x*_‐NSs 1:5 blend (red); b) Transmittance spectra of NSs (blue), Ti_3_C_2_T_*x*_(black) and Ti_3_C_2_T_*x*_‐NSs 1:5 blend (red); c) XPS Ti 2p spectra of Ti_3_C_2_T_*x*_ and Ti_3_C_2_T_*x*_‐NSs composites; NSs spectra at the same binding energy range is reported as reference; d) XRD patterns of Ti_3_C_2_T_*x*_, NSs and Ti_3_C_2_T_*x*_‐NSs 1:5 composites.

**Figure** **2**a shows the Raman spectra (in the range of 120–800 cm^−1^) of Ti_3_C_2_T_*x*_ MXene (black curve), NSs (blue curve) and the Ti_3_C_2_T_*x*_‐NSs (1:5 weight ratio) samples (red curve). The Raman spectrum of the Ti_3_C_2_T_*x*_ MXenes shows two broad resonant regions, denoted as the T_*x*_ (functional groups) region (between ≈223 and ≈480 cm^−1^) and the C region (between ≈503 and ≈750 cm^−1^).^[^ ^24^ ^]^ The convoluted peaks in the T_*x*_ region originate from the *E*_g_ in‐plane vibrations of the T_*x*_, while the convoluted peaks in the C region are caused by both in‐plane *E*_g_ vibrations and A_1g_ out‐of‐plane vibrations of the carbon atoms. An additional peak appears at ≈205 cm^−1^ originates from the A_1g_ out‐of‐plane vibrational mode of the Ti atoms and T_*x*_ (—OH, —O, and —F^[^ ^25^ ^]^). NSs are composed of a mixed ZnOHF‐ZnO phase. The Raman spectrum of NS (blue curve) shows resonant peaks at about 158, 181, and 366 cm^−1^,^[^ ^26^ ^]^ and a sharp but less intense peak at 725 cm^−1^, which comes from the Zn(OH)_2_ residuals.^[^ ^26^ ^]^ The peak at 158 cm^−1^ is attributed to the ZnO 2E_2L_ and the one at 181 cm^−1^ corresponds to the ZnO 2E_2_ peak. The strong feature at 366 cm^−1^ is coming from the ZnOHF structure.^[^ ^26^ ^]^ A broad and weak shoulder can be identified at 432 cm^−1^ which is assigned to the ZnO E_2_ mode, which is the characteristic of the wurtzite hexagonal phase of ZnO.^[^ ^27^ ^]^ The Raman spectrum of the Ti_3_C_2_T_*x*_‐NSs 1:5 sample contains resonant peaks coming from both the ZnOHF‐ZnO NSs and the Ti_3_C_2_T_*x*_ MXene, as expected. Some NSs peaks are hidden by the broad‐resonant Raman peak regions of MXene (e.g., the 725 cm^−1^ peak falls in the MXene C region, while the 366 cm^−1^ and 432 cm^−1^ weak peaks are shadowed by the T_*x*_ region), while the A_1g_ peak in the MXene is convoluted with the ZnO 2E_2_ peak at 181 cm^−1^. Black and blue dashed lines are a guide to the eye to identify the main peaks belonging to Ti_3_C_2_T_*x*_ and NSs, respectively. Figure 2b represents the transmittance, T(%), spectra as a function of the wavelength (*λ*) (in the range of 200–1100 nm) of a film, obtained by drop casting the sample solution on quartz slides, of NSs (blue) and Ti_3_C_2_T_*x*_‐NSs 1:5 (red curve). For NSs, a significant absorption in the ultraviolet range, as the % transmittance goes to zero. The NSs curve shows a typical T for NSs with an onset at ≈300 nm.^[^ ^28^ ^]^ The Ti_3_C_2_T_*x*_‐NSs 1:5 shows a broad T spectrum, going to zero at about 330 nm, indicating a lower optical band gap (*E*_g_) for this material.^[^ ^29^ ^]^ We used the Tauc plot to extract the optical bandgap (*E*_g_) of the NSs obtaining *E*_g_ ≈ 3.02 eV, as the Tauc plot is a method originally developed to derive the optical gap of semiconductors. The Ti_3_C_2_T_*x*_‐NSs 1:5 is composed of a mixture of Ti_3_C_2_T_*x*_ flakes evidencing a metallic behavior,^[^ ^24^, ^30^ ^]^ and ZnO NSs which are a semiconducting material,^[^ ^29^ ^]^ therefore making the Tauc method not a viable route to estimate E_g_ in this composite material.^[^ ^31^ ^]^ Figure 2c shows the high‐resolution XPS spectra (over the 449‐ 472 eV region) of the Ti 2*p* region for Ti_3_C_2_T_*x*_ (empty black circles), NSs (empty blue circles), and the Ti_3_C_2_T_*x*_‐NSs 1:5 (empty red circles). The Ti 2*p* region in Ti_3_C_2_T_*x*_ MXene comprises of three doublet peaks. The Ti(II) and Ti(III) peaks are attributed to the formation of T_*x*_.^[^ ^32^ ^]^ The asymmetric Ti—C (Ti 2*p*3 ≈ 454.7 eV and Ti 2*p*1 ≈ 460.0 eV) peaks are shifted to a larger binding energy compared with its precursor Ti_3_AlC_2_ phase (Ti—C 2*p*3 ≈ 454.6 eV),^[^ ^33^ ^]^ which is attributed to the Al atom being replaced by more electronegative termination. The XPS survey spectra for Ti_3_C_2_T_*x*_‐NSs 1:5 and NSs, and the high‐resolution XPS spectra of the Zn 2*p* and O 1*s* regions for NSs are reported in Figure S2, Supporting Information. The doublet peaks featuring in the Ti 2*p* region of the Ti_3_C_2_T_*x*_‐NSs 1:5 spectrum, confirm the presence of Ti_3_C_2_T_*x*_ flakes in the composite. Figure 2d represents the XRD patterns of the Ti_3_C_2_T_*x*_ (black curve), NSs (blue curve), and the Ti_3_C_2_T_*x*_‐NSs 1:5 (red curve). The black curve shows peaks at 2*θ* = 7.2°, 14.5° and 29°, assigned to the (002), (004), and (008) plane diffractions. The (002) peak at 2*θ* = 7.2° is downshifted compared to that of Ti_3_AlC_2_ (2*θ* = 10°),^[^ ^33^ ^]^ which indicates the successful etching of the Al atom and delamination of the MXene.^[^ ^33^ ^]^ The XRD spectrum of the NSs sample (blue curve) possesses both ZnO (black labels) and ZnOHF (red labels) peaks (PDF Cards No. 00‐036‐1451 and No. 74‐1816, respectively), in line with previous reports.^[^ ^34^ ^]^ The spectrum of the Ti_3_C_2_T_*x*_‐NSs 1:5 composite (red curve), presents peaks assigned to both Ti_3_C_2_T_*x*_ and NSs, confirming the presence of both materials.

Cyclic voltammetry (CV) measurements are the best technique to find the potential window of interest for electrodes in energy storage applications. We performed CV measurements on electrodes prepared from our Ti_3_C_2_T_*x*_‐NSs composite material, comparing it with MXene and NSs reference electrodes, to assess the storage properties of our material, and evaluate their suitability for supercapacitors.

**Figure 3:**
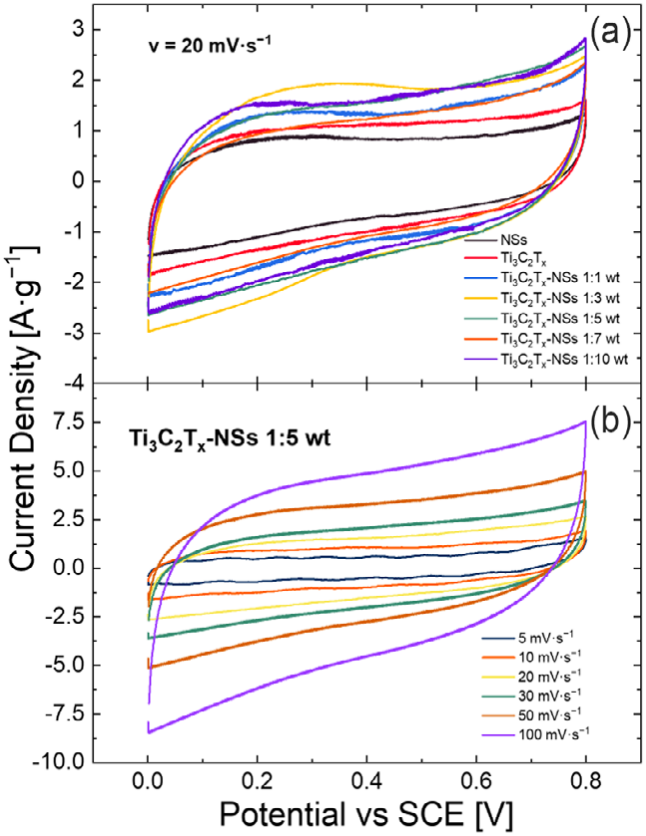
a) CV curves at 20 mV s^−1^ in 1 M Na_2_SO_4_ of NSs (blue curve), Ti_3_C_2_T_*x*_ (black curves) and MXene‐NSs mixture (1:1 min, 1:3 orange, 1:5 red, 1:7 yellow, 1:10 pink), and b) CV curves for Ti_3_C_2_T_*x*_‐NSs 1:5 mixture at different scan rates.

**Figure** **3**a shows the CV curves of spray‐coated electrodes prepared from Ti_3_C_2_T_*x*_‐NSs dispersions with different weight ratios at a scan rate (*v*) of 20 mV s^−1^. Ti_3_C_2_T_*x*_ and NSs curves (black and blue curves, respectively) are reported as reference. The curves of all Ti_3_C_2_T_*x*_‐NS electrodes show greater CV areas than that of Ti_3_C_2_T_*x*_ and NSs references, meaning that a greater amount of charge was stored within each CV cycle. Also, the more regular CV shapes of NSs and MXene curves are typical of capacitive materials.^[^ ^35^ ^]^ Figure 3b shows the CV curves acquired for a Ti_3_C_2_T_*x*_‐NSs electrode (1:5 weight ratio) in a 1M Na_2_SO_4_ solution at different scan rates, from 5 to 100 mV s^−1^, while CV curves for the other blends (1:1, 1:3, 1:7, and 1:10) are reported in Figure S3, Supporting Information.

**Figure 4:**
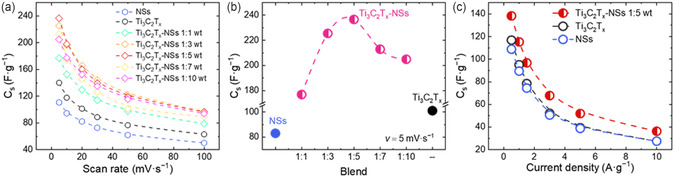
a) *C*_s_ as a function of scan rates for all samples analyzed; b) *C*_s_ from CV curves at a scan rate of 5 mV s^−1^ for all blends, Ti_3_C_2_T_*x*_ and NSs, and c) *C*_s_ for Ti_3_C_2_T_*x*_‐NSs 1:5, Ti_3_C_2_T_*x*_ and NSs as a function of current densities, obtained from GCDs.

From the CV curves, the specific capacitance (*C*_s_) values can be extrapolated, specifying which sample possesses the best performance. **Figure** **4**a summarizes the *C*_s_ values as a function of *v* for Ti_3_C_2_T_*x*_, Ns and Ti_3_C_2_T_*x*_‐NSs. For all the samples, a marked dependence on the scan rate was observed, with the best sample, Ti_3_C_2_T_*x*_‐NSs (1:5 weight ratio) having the highest *C*_s_ value at 236 F g^−1^ at 5 mV s^−1^. Considering the CV shapes and the *C*_s_ results for all samples, the total charge stored of the cathode can be originated from two components: a Faradaic contribution from charge‐transfer processes of the ions of the electrolyte with surface atoms at the electrode interface, referred to as pseudocapacitance (typically showing strong dependence of the *C*_s_ on the scan rate); and a non‐Faradaic contribution from the double‐layer effect (typically there's no dependence of performances on the scan rate).^[^ ^34^, ^35^ ^]^ Figure 4b shows the *C*_s_ from CVs curves at a scan rate of 5 mV s^−1^ for the Ti_3_C_2_T_*x*_‐NSs electrodes (pink). The *C*_s_ values of Ti_3_C_2_T_*x*_ (black dot) and NSs (blue dot) are also reported as references. The *C*_s_ values exhibited a clear bell‐shaped trend as a function of the Ti_3_C_2_T_*x*_‐NSs weight ratio (from 1:1 to 1:10), with a maximum of 236 F g^−1^ at 5 mV s^−1^ at the 1:5 weight ratio. Noteworthy, all Ti_3_C_2_T_*x*_‐NSs electrodes possess a *C*_s_ greater than the NSs (107 F g^−1^ at 5 mV s^−1^) and Ti_3_C_2_T_*x*_ MXene (140 F g^−1^ at 5 mV s^−1^) electrodes. We adopted GCD measurements to obtain more accurate results in terms of performances (*C*_s_).^[^ ^36^ ^]^ Figure S4a–c, Supporting Information compares the GCD curves for the Ti_3_C_2_T_*x*_‐NSs (1:5 weight ratio), Ti_3_C_2_T_*x*_ and NSs electrodes. The calculated *C*_s_ of the Ti_3_C_2_T_*x*_‐NSs 1:5 electrode from GCD curves (Figure 4c) results is the highest at any current density (0.5 to 10 A g^−1^), going from 139 F g^−1^ at 0.5 A g^−1^ to 37 F g^−1^ at 10 A g^−1^ compared to NSs and the Ti_3_C_2_T_*x*_ electrodes, showing a *C*_s_ peaking at 102 F g^−1^ at 0.5 A g^−1^ and 110 F g^−1^ at 0.5 A g^−1^, respectively, hence, confirming a higher *C*_s_ in the Ti_3_C_2_T_*x*_‐NSs electrodes.

**Figure 5:**
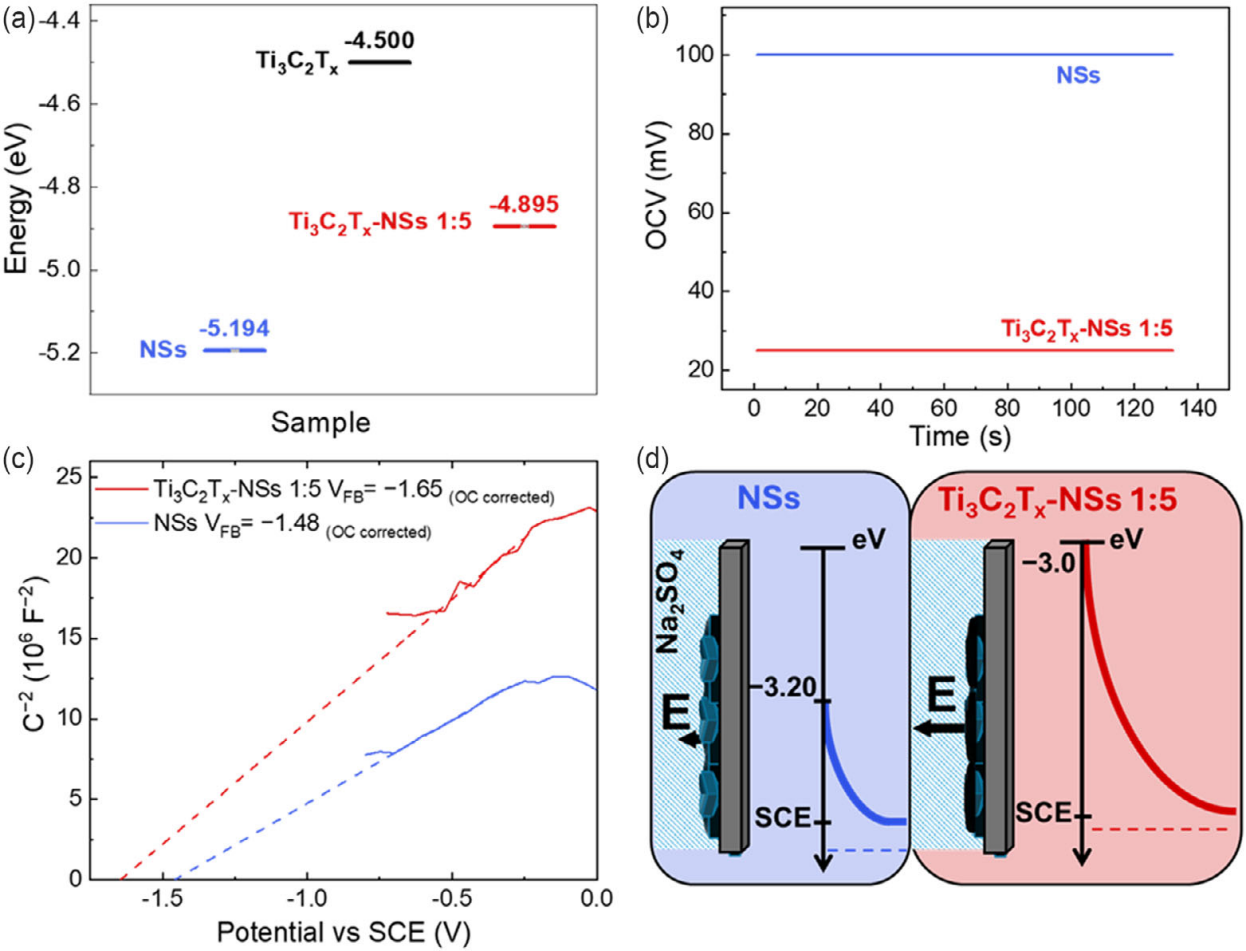
a) Fermi energy of NSs, Ti_3_C_2_T_*x*_ and Ti_3_C_2_T_*x*_‐NSs 1:5; b) open‐circuit voltage for NSs and Ti_3_C_2_T_*x*_‐NSs 1:5 in 1M Na_2_SO_4_ c) NSs and Ti_3_C_2_T_*x*_‐NSs 1:5 Mott−Schottky plots; and d) the band bending schematic, with the generated electric field at the electrode/electrolyte interface.

To gain further insights into such a higher *C*_s_ value, we used Kelvin probe microscopy, open circuit voltage (OCV), and Mott–Schottky analyses to obtain the flat band potential, and the electric field (E) at the electrode/electrolyte interface in the NSs and Ti_3_C_2_T_*x*_‐NSs 1:5 electrodes. **Figure** **5**a represents the Fermi energies (*E*_F_) acquired for NSs, Ti_3_C_2_T_*x*_, and Ti_3_C_2_T_*x*_‐NSs 1:5 electrodes. The Ti_3_C_2_T_*x*_ and the NS electrodes show *E*_F_ ≈ 4.5 eV and *E*_F_ ≈ −5.2 eV, respectively. The Ti_3_C_2_T_*x*_‐NSs 1:5 electrode shows *E*_F_ ≈ −4.9 eV which falls within the *E*_F_ range of the two initial materials, revealing a good combination of Ti_3_C_2_T_*x*_ and NSs as expected.^[^ ^37^ ^]^ The resultant Fermi level reflects the balance of electronic states contributed by both materials and a better n‐type conductivity is obtained in the blend system compared with bare NSs. With shallower Fermi level in this blend system, the difference of Fermi level between Ti_3_C_2_T_*x*_‐NSs 1:5 electrode and Na_2_SO_4_ electrolyte is larger than the case of bare NSs, leading to a larger band bending at the interface, which is beneficial to electron and negative ions transport towards the electrode. Figure 5b reports the OCV for both electrodes in 1M Na_2_SO_4_ electrolyte. They present a positive OCV, with NSs having an OCV of 100 mV, while the Ti_3_C_2_T_*x*_‐NSs 1:5 possesses an OCV of 25 mV. A Mott–Schottky plot for NSs and Ti_3_C_2_T_*x*_‐NSs 1:5 is reported in Figure 5c, where the dashed line is the projection of the plot linear region. A standard Mott–Schottky plot depicts the relationship between the reciprocal of squared capacitance (C^−2^) and the applied potential on a sample.^[^ ^38^, ^39^ ^]^ In the case of an n‐type semiconductor like ZnO, a decrease in the applied potential causes C^−2^ to approach the zero value.^[^ ^38^, ^39^ ^]^ The potential at which this happens (i.e., where the plot intersects the x‐axis) represents the flat‐band potential (*V*_FB_), which needs to be adjusted for the open circuit voltage to obtain $\cdot V_{M-S}=V_{\text{FB}}-OCV$.^[^ ^40^ ^]^ ZnO is an n‐type semiconductor, and when submerged in an electrolytic solution, it balances the carriers at the solid–liquid interface by aligning the Fermi energy of the material with the redox potential of the electrolyte.^[^ ^39^ ^]^ This process depletes the semiconductor of electrons, leading to an accumulation of holes beneath its surface and an upward bending of the semiconductor energy bands.^[^ ^39^ ^]^ By controlling the applied voltage, it is possible to manipulate the extent of energy level bending.^[^ ^41^ ^]^ The flat‐band potential, *V*_FB_, is achieved when the applied potential is sufficient to flatten the semiconductor bands, regardless of the redox potential position in the solution. The introduction of Ti_3_C_2_T_*x*_ flakes clearly influence the position of NSs energy levels at the interface of the Ti_3_C_2_T_*x*_‐NSs electrode. As demonstrated by the Mott–Schottky analyses, the *V*_FB_ on the Ti_3_C_2_T_*x*_‐NSs 1:5 electrode (−1.65 V) is nearly 200 mV higher than that of the NS electrode (−1.48 V). This suggests that the interface between the metallic MXene and the semiconducting ZnO facilitates the formation of ion transport channels, likely by modifying the surface charge density and increasing chemical reactivity.^[^ ^42^ ^]^

Figure 5d sketches the band bending for both NSs and Ti_3_C_2_T_*x*_‐NSs 1:5 electrodes, where a saturated calomel electrode (SCE, 4.68 eV) represents the absolute electrode potential.^[^ ^43^ ^]^ By subtracting the *V*_FB_ values for both NSs or Ti_3_C_2_T_*x*_‐NSs 1:5 with respect to the absolute electrode potential we obtained a band bending of Ti_3_C_2_T_*x*_‐NSs 1:5–3.0 eV and a band bending for NSs–3.2 eV, respectively. Dashed lines represent the OCV values for both Ti_3_C_2_T_*x*_‐NSs 1:5 and NSs samples.

This difference can be explained considering that the presence of Ti_3_C_2_T_*x*_ nanoflakes attracts electrons from the ZnO at the interface, a phenomenon known as “spillover”,^[^ ^42^ ^]^ hindering the flow of electrons between the electrode and the electrolyte and creating holes at the nanostructure surface. An electric field pointing outside (towards the electrolyte) will be formed, and, considering these band schemes, the electric field on the Ti_3_C_2_T_*x*_‐NSs 1:5 electrode will be stronger than the NSs electrode, thus attracting more negative ions and increasing the stored charge at the electrode interface (black rows in the schematic at the electrode/electrolyte interface).^[^ ^44^ ^]^

**Figure 6:**
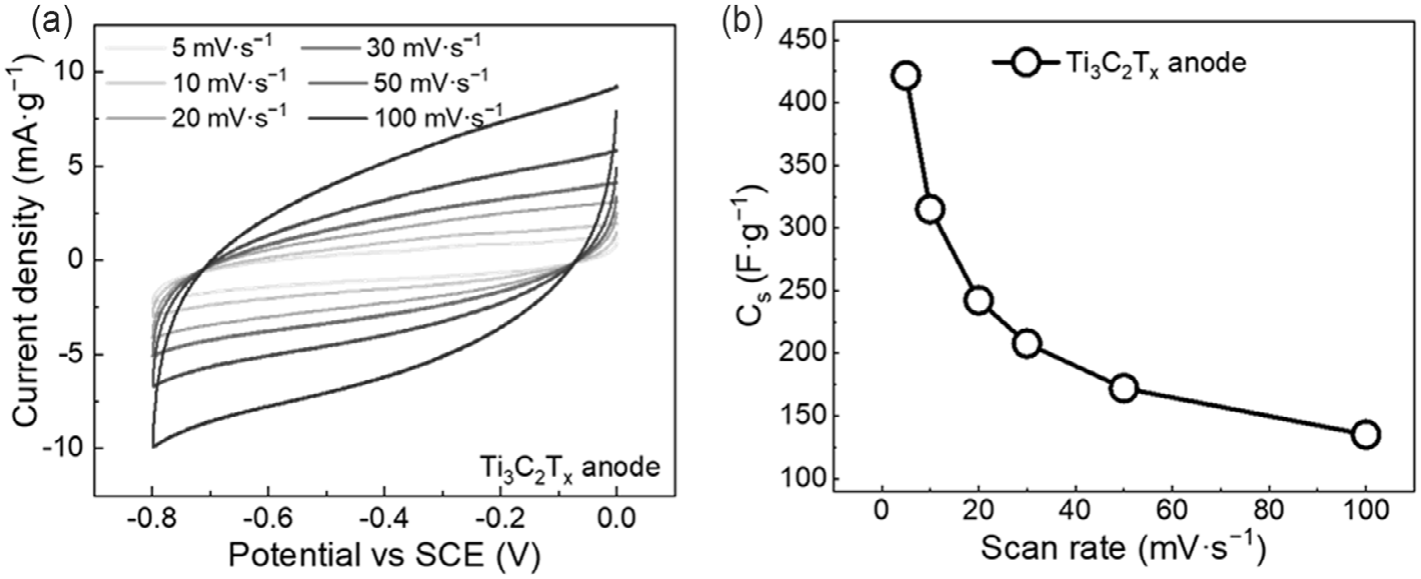
a) CV curves for Ti_3_C_2_T_*x*_ anode at different scan rates, and b) anode C_*s*_ at different scan rates.

We evaluate the performance of the Ti_3_C_2_T_*x*_‐NSs 1:5 in a 2‐electrode aqueous asymmetric supercapacitor device (ASC) prototype. A bare Ti_3_C_2_T_*x*_ anode was prepared by spray coating (mass of 0.2 mg) and characterized in the mirror cathode potential window. All the other electrochemical cell characteristics (reference electrode, counter electrode, and electrolyte) remain the same depicted in Figure 1a. **Figure** **6**a shows the CV curves of the Ti_3_C_2_T_*x*_ anode as a function of the scan rate, and Figure 6b reports the related *C*_s_, obtained from CV curves. A mass‐balanced cell should be considered to maximize the overall performance of the supercapacitors. From the *C*_s_ values, it is possible to balance the mass of the cathode and anode electrodes and couple them in a device.^[^ ^45^ ^]^ The charge (*Q*) stored is related to the *C*_s_, the Δ*V* and the active mass (*m*) of the corresponding electrode, anode (−), and cathode (+):^[^ ^45^ ^]^

$$m_{+}\Delta VC_{s+}=m_{-}\Delta VC_{s-}$$

Considering an active mass of 0.2 mg for the anode ($m_{-})$, the Δ*V* as 0.8 V and the *C*_s_ values at 5 mV s^−1^ for both cathode and anode electrodes, we obtain that the cathode active mass needs to weigh $m_{+}$ ≈ 0.36 mg.

**Figure 7:**
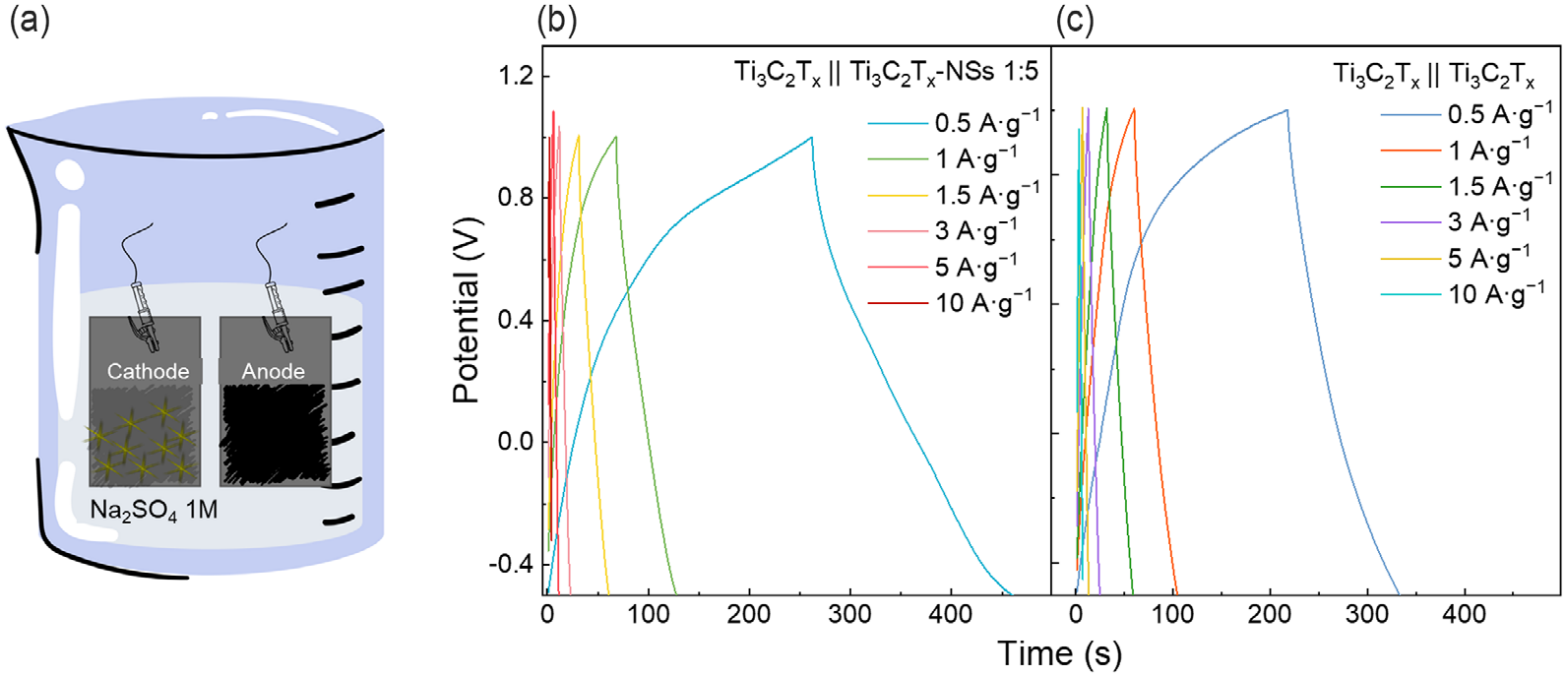
a) device schematic for the Ti_3_C_2_T_*x*_|| Ti_3_C_2_T_*x*_‐NSs 1:5 cell b) CV curves for Ti_3_C_2_T_*x*_||Ti_3_C_2_T_*x*_‐NSs 1:5 and c) Ti_3_C_2_T_*x*_||Ti_3_C_2_T_*x*_ devices at different scan rates in 1M Na_2_SO_4_.

**Figure** **7**a shows the ASC scheme for the Ti_3_C_2_T_*x*_ || Ti_3_C_2_T_*x*_‐NSs 1:5 (anode || cathode). The ASC was tested in 1M aqueous Na_2_SO_4_ in the −0.5 ÷ 1 V potential region (see the Materials and Methods section for more details). A Ti_3_C_2_T_*x*_ || Ti_3_C_2_T_*x*_ symmetric device was prepared for performance comparison. Figures 7(b,c) show the GCD curves for the ASC and the symmetric device. Charge and discharge curves are both different for the two devices reported. Indeed, the ASC shows typical pseudocapacitive behavior, as evidenced by the change in slope of the GCD charge and discharge curves, while that of the symmetric Ti_3_C_2_T_*x*_ || Ti_3_C_2_T_*x*_ device is more triangular, typical of capacitive materials.^[^ ^6^ ^]^ Our cathode Ti_3_C_2_T_*x*_‐NSs 1:5 has already proven its pseudocapacitive behavior, as confirmed by this GCD. It is also evident that the discharge time of the Ti_3_C_2_T_*x*_ || Ti_3_C_2_T_*x*_ symmetric device (118 s) is shorter than the ASC one (199 s) by 81 s, resulting in a higher storage capacity of the Ti_3_C_2_T_*x*_ || Ti_3_C_2_T_*x*_‐NSs 1:5 device.

**Figure 8:**
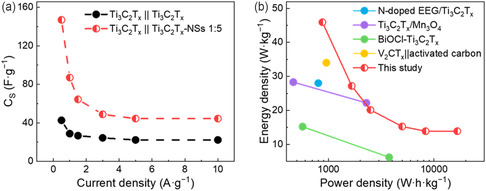
a) C_s_ for Ti_3_C_2_T_*x*_||Ti_3_C_2_T_*x*_‐NSs 1:5 and Ti_3_C_2_T_*x*_||Ti_3_C_2_T_*x*_ devices as a function of current densities, obtained from GCDs and b) Ragone plot obtained from the as tested device (half circles) compared to those reported in the literature for MXene‐metal oxide/MXene devices.

**Figure** **8**a compared the *C*_s_ obtained from the GCD curves for both the symmetric Ti_3_C_2_T_*x*_ || Ti_3_C_2_T_*x*_ device (black line and dots) and the ASC (red line and dots) as a function of current densities. The ASC Ti_3_C_2_T_*x*_ || Ti_3_C_2_T_*x*_‐NSs 1:5 device reports *C*_s_ ≈ 147 F g^−1^ compared to *C*_s_–42 F g^−1^ of the symmetric Ti_3_C_2_T_*x*_ || Ti_3_C_2_T_*x*_ device, which is a remarkable value considering that the device is working in a neutral pH, where corrosion phenomena due to water splitting reactions are circumvented,^[^ ^7^ ^]^ highlighting the prospects that this device possesses. From GCD, *E*_d_ and *P*_d_ were calculated as reported in the material and methods section. The Ragone plot in Figure 8b shows the data from this work (red lines and half circles) compared to other relevant works (all other lines with full circles symbols). The Ti_3_C_2_T_*x*_ || Ti_3_C_2_T_*x*_‐NSs 1:5 device shows the highest *E*_d_ ≈ 46 W h kg^−1^ at *P*_d_ ≈ 875 W kg^−1^ and the highest *P*_d_ ≈ 16 650 W kg^−1^ at *E*_d_ ≈ 14 W h kg^−1^.^[^ ^46^, ^47^, ^48^, ^49^ ^]^ Noteworthy in the Ragone plot, batteries occupy the upper left corner, capacitors are situated in the lower right corner, and pseudocapacitors serve as a bridge between the two. Our data cover an extensive range of performances, featuring numerous experimental points, a crucial aspect for maximizing *E*_d_ and *P*_d_. Also, the values obtained are higher than those reported for supercapacitors operating in neutral pH, such as the V_2_CT_*x*_ MXene || activated carbon (yellow point, 34 W h kg^−1^ at 954 W kg^−1^ ZnSO_4_ electrolyte),^[^ ^46^ ^]^ BiOCl‐Ti_3_C_2_T_*x*_ (green line‐symbols, 15.2 W h kg^−1^ at 567.4 W and 6.2 W h kg^−1^ at 3756.8 W kg^−1^, 1M KOH electrolyte),^[^ ^47^ ^]^ Ti_3_C_2_T_*x*_‐Mn_3_O_4_ (violet line‐symbols, 28.3 W h kg^−1^ at 463.4 W kg^−1^ and 22.2 W h kg^−1^ at 2285.5 W kg^−1^, 6M KOH electrolyte),^[^ ^48^ ^]^ and N‐doped EEG/ Ti_3_C_2_T_*x*_ (28 W h kg^−1^ at 800 W kg^−1^, 2.5M KNO_3_).^[^ ^49^ ^]^ Our results are a direct consequence of the increased electric field at the electrode/electrolyte thanks to the engineered Ti_3_C_2_T_*x*_‐NSs composite electrode. This improves the electrochemical performance of the whole device in terms of energy storage. Our ASC Ti_3_C_2_T_*x*_ || Ti_3_C_2_T_*x*_‐NSs 1:5, working at neutral media, is very competitive with respect to other aqueous devices working at more extreme pH, keeping all the advantages of a device working at neutral pH mentioned above. As shown in the Ragone plot, our device is able to range in energy density and power density ranges not yet explored by this type of materials, paving the way to a whole series of future studies. This device could potentially be used in the future as a viable energy storage solution.

## 3 Conclusions

This work demonstrates a high‐performance Ti_3_C_2_T_*x*_‐NSs cathode electrode for energy storage applications. Different blends for the electrode material were prepared via spray coating and the obtained electrodes were tested as cathode in neutral pH (1M Na_2_SO_4_). The Ti_3_C_2_T_*x*_‐NSs 1:5 blend showed the best performances in terms of specific capacitance, with a *C*_s_ of 236 F g^−1^ at 5 mV s^−1^ (CV) and of 139 F g^−1^ at 0.5 A g^−1^ (GCD). A synergistic enhancement in performance is expected, harnessing the combined advantages of both materials. This is due to the increased electric field by the blends pointing towards the electrolyte, which attracts an increased number of negative ions, therefore increasing the charge stored at the electrode–electrolyte interface, as demonstrated by the Mott–Schottky analysis. A Ti_3_C_2_T_*x*_ anode electrode was prepared and tested, and an asymmetric supercapacitor device Ti_3_C_2_T_*x*_||Ti_3_C_2_T_*x*_‐NSs 1:5 device was tested in neutral pH, showing a *C*_s_ of 147 F g^−1^ at 0.5 A g^−1^, the highest *E*_d_ of 46 W h kg^−1^ at a *P*_d_ of 875 W kg^−1^ and the highest *P*_d_ of 16 650 W kg^−1^ at an *E*_d_ of 14 W h kg^−1^. Compared to other MXene‐metal oxide devices in neutral or alkaline media, our electrode presents the best performances in terms of both energy and power densities. All the results suggest that Ti_3_C_2_T_*x*_ MXene boosts promising nanomaterials such as ZnO NSs in efficient energy storage devices owing to the synergy between the double‐layer capacitance and the pseudocapacitive effect.

## 4 Experimental Section

4.1

4.1.1

##### Materials and Methods

The detailed procedure for preparing NSs through chemical bath deposition followed the protocol detailed in ref. [34] NSs were synthesized via chemical bath deposition (CBD) using zinc nitrate, HMTA, and ammonium fluoride. The solutions were heated to 90 °C, mixed in sequence, and maintained at 90 °C for 10 min.^[^ ^28^, ^34^ ^]^ NSs were thoroughly washed with deionized water (MilliQ) through decantation and then dried in oven (air environment) at 100 °C for 16 h to remove water residuals. A 2 mg mL^−1^ NS solution was prepared by weighing 2 mg of NSs obtained powders and dispersing the NSs powders in 1 mL of deionized water through sonication for 5 min. The Ti_3_C_2_T_*x*_ MXene was synthesized from MAX phase (Ti_3_AlC_2_) powder (Carbon‐Ukraine Ltd.) using in situ HF through addition of lithium fluoride (LiF, Thermo Scientific) into hydrochloric acid (HCl, 37%, Fisher Scientific), which is a simplified and safer synthesis method improving MXene performance in energy storage applications.^[^ ^50^, ^51^ ^]^ As reported in ref. [33] 14 mL HCl (12 M) was added into 6 mL deionized water in a vented polyethylene (HDPE) container, followed by adding 1.5 g LiF powder into the solution. The mixture was stirred for 5 mins at room temperature using a magnetic polytetrafluoroethylene (PTFE) bar to fully dissolve the salt. MAX powder (1 g) was then carefully added to the solution over the course of 10 mins to avoid initial overheating by aggressive exothermal reactions. The container was then immersed in a silicon oil bath at 45 °C for 24 h to allow a fully selective aluminum etching. The fully etched mixture was washed with deionized water for 4–5 cycles via centrifugation (GT2R centrifuge, Fisher, TX‐400 rotor) until the pH of the supernatant reached ≈6. The fully washed mixture was redispersed in deionized water and manually shook^[^ ^52^ ^]^ to enable delamination of MXene flakes. After centrifugation at 800 rcf for 10 min, the supernatant was then collected as the Ti_3_C_2_T_*x*_ MXene dispersion. The Ti_3_C_2_T_*x*_‐NSs dispersions were prepared by mixing different aliquots of a 2 mg mL^−1^ Ti_3_C_2_T_*x*_ MXene dispersion and a 2 mg mL^−1^ NS dispersion at different weight ratios (1:1, 1:3, 1:5, 1:7, and 1:10) followed by 10 min sonication to allow homogeneous blends. A polyvinylidene fluoride (PVDF) solution in acetone was then added as binder^[^ ^53^ ^]^ to the Ti_3_C_2_T_*x*_‐NSs dispersions (10% v/v). The electrodes were then prepared by spray coating the different (Ti_3_C_2_T_*x*_, NSs and Ti_3_C_2_T_*x*_‐NSs) dispersions separately onto graphene paper (GP) substrates (240 μm thick, Sigma Aldrich, St. Louis, MO, USA) and dried under nitrogen flux. The final electrodes had a loaded mass of 0.2 mg, measured with a Mettler Toledo (Columbus, OH, USA) MX5 Microbalance (sensitivity: 0.01 mg). The Ti_3_C_2_T_*x*_‐NSs electrode with a 1:5 weight ratio was the one selected for the morphological, optical, and spectroscopy characterization.

##### 4.1.1.1 Morphological, Optical, and Electrochemical Characterization

Scanning electron microscopy (Gemini field emission SEM Carl Zeiss SUPRA 25, Carl Zeiss Microscopy GmbH, Jena, Germany) was used to study the surface morphology of the NSs and the Ti_3_C_2_T_*x*_ flakes. The measurements were conducted with an acceleration voltage ranging from 3 to 5 kV. The aperture size was set to 30 μm, and the working distance ranged from 2 to 5 mm. An in‐lens detector was used to enhance the signal from secondary electrons (SE) generated in the upper part of the interaction volume and low‐loss backscattered electrons from the beam spot center. This setup allowed us to obtain high‐contrast images that provide direct information about the sample surface. Statistical analysis of the SEM images was performed using the Gatan Digital Microscope software. SEM images were analyzed using ImageJ software to improve the brightness and contrast.^[^ ^54^ ^]^

Atomic force microscopy (AFM, Keysight 5500 SPM AFM, Keysight Technologies, California, the United States) analysis was performed on the Ti_3_C_2_T_*x*_ MXene flakes to reveal flake thickness and lateral size distribution. Measurements were collected over a 20 μm × 20 μm area at a scanning rate of 0.40 Hz using a point probe (Silicon‐SPM‐Sensor, PPP‐NCLR‐50, Nano‐sensors) operating in a tapping mode. The dispersions were diluted by a factor of 1000 and then drop casted onto a precleaned Si/SiO_2_ substrate. Statistical analysis over 89 flakes was carried out to reveal the thickness (<*t*>) distribution and lateral size <*S>* distribution of the Ti_3_C_2_T_*x*_ MXene flakes. The <*S>* is obtained via <*S> = xy* ^0.5^, where *x* and *y* refer to the length and width of a particular flake. To determine the Fermi level (*E*_F_) of the samples, an APS04 (KP Technology) with a vibrating tip (gold, 2 mm) Kelvin probe was applied. The controlled average temperature was 20.6 °C (±0.2 °C) and the relative humidity was 33.1% (±3.4%). The contact potential difference (CPD) between the sample and the tip was collected from the steady state of signal. The work function was calculated by adding the tip's work function to the final equilibrium value of samples. In order to determine the tip's work function, the CPD of a freshly cleaned silver reference was measured. Fermi energy is equal to the work function in the dark.

Raman characterization (WiRe 4.1, inVia micro‐Raman spectrometer, Renishaw, England, UK) was acquired to examine the material quality. A 532nm laser with a × 50 objective lens was applied and an incident power of ≤1 mW was used to avoid thermal damage. The samples for Raman spectroscopy were drop cast as thin films on precleaned (washed by iso‐propanol/acetone) Si/SiO_2_ substrates (Si‐Mat). The X‐ray photoelectron spectroscopy (XPS, Axis Supra system, Kratos Analytical, Manchester, UK) spectra were acquired to examine the stoichiometry of the materials using an Al *α* X‐ray source focused into an 800 μm spot. The XPS samples were drop cast as thin films on precleaned glass. The data analysis was performed in Avantage 5.99 (Thermo Scientific). X‐ray powder diffraction (XRD, D8 advance diffractometer, AXS system, Bruker, Massachusetts, USA) patterns were acquired to examine the crystallinity change of materials. Measurements were scanned within a 2*θ* range from 5° to 70° at a step scan of 0.02°, with a fixed slit size of 0.6 mm. The XRD samples were deposited as thin films on precleaned glass. Optical measurements were carried out using a LAMBDA 1050 + UV/Vis/NIR spectrometer along with 150 mm integrating sphere (PerkinElmer, Inc., Shelton, CT USA). Electrochemical measurements of the electrodes were carried out at room temperature using a VersaSTAT4 potentiostat (Princeton Applied Research, Oak Ridge, TN, USA), and a three‐electrode setup (Figure 1a) was used with a platinum wire as a counter electrode, a saturated calomel electrode (SCE) as a reference electrode, and the Ti_3_C_2_T_*x*_:NS samples on GP substrates as a working electrode (WE). 1M Na_2_SO_4_ solution (Sigma Aldrich, St. Louis, MO, USA, ≥85%) was used as the supporting electrolyte. The neutral electrolyte was chosen to avoid corrosion problems related to water splitting reactions.^[^ ^7^ ^]^ CV and GCD analyses were conducted in the potential range 0 ÷ 0.8 V versus SCE for the cathode electrode (Ti_3_C_2_T_*x*_‐NSs 1:5). CV curves were recorded at different scan rates (5 to 100 mV s^−1^), and GCD tests were conducted at different current densities (0.5 to 10 A g^−1^).

The specific capacitance (*C*_s_) of each electrode can be obtained from the area contained within the CV curve, calculated as follows.^[^ ^34^, ^55^ ^]^

$$C_{s}=\frac{\int I\text{dV}}{m\upsilon \Delta V}$$

where *I* is the measured current (mA), *m* is the active mass (mg), *υ* is the voltage scan rate (mV s^−1^), and ΔV is the potential range (V). We also obtained the *C*_s_ from the discharge time of the GCD measurements using the formula below

$$C_{s,\text{GCD}}=\frac{It_{s}}{m\Delta V}$$

where $t_{s}$ is the discharge time (s), *I* is the applied current (mA), ΔV is the voltage range (V), and *m* is the active electrode (NSs, Ti_3_C_2_T_*x*_, or the blends) mass (mg). The electrochemical measurements in a two‐electrode setup were performed with a Metrohm Autolab PGSTAT204 potentiostat controlled by NOVA 2.1.6 software (Methrom Autolab B. V., The Netherlands) in the potential range −0.5 ÷ 1 V. The energy [W h kg^−1^] and power densities [W kg^−1^] were derived by GCD analysis via the following equations^[^ ^55^ ^]^

$$E_{d}=\frac{1}{2\Delta 3.6}C_{s,\mathrm{GCD}}\Delta V^{2}$$

$$P_{d}=E_{d}\cdot \frac{3600}{\Delta t}$$

where $C_{s,\text{GCD}}$ is the specific capacitance obtained from GCD curves, ΔV is the potential range (1.5 V), Δt is the discharge time and the coefficients 3.6 and 3600 are unit measurements conversions (from seconds and grams to hours and kilos, respectively).

## Conflict of Interest

The authors declare no conflict of interest.

## Supporting information

Supplementary Material

## Data Availability

Research data are not shared.
