# Supplementary material for: Advanced Pseudocapacitive Performances of a Ti3C2Tx–ZnOHF/ZnO Nanocomposite for Energy Storage Applications
Source: ChemSusChem. 2025 Jul 8;18(15):e202500024. doi: 10.1002/cssc.202500024 (PMC12302305; doi:10.1002/cssc.202500024)
Supplement: Supplementary file 1 — Supplementary Material [file CSSC-18-e202500024-s001.pdf]

Supporting Information

Advanced pseudocapacitive performances of a $Ti_3C_2T_x$-ZnOHF/ZnO nanocomposite for energy storage applications

*Gisella M. Di Mari, Chengning Yao; Tianhao Lan; Sihui Liu, Giacometta Mineo; Vincenzina Strano; Elena Bruno; Ji-seon Kim; Salvatore Mirabella; Felice Torrisi**

AFM statistics reported in Figure S1(a) was used to estimate the average thickness (<*t*>) and lateral size (<*S*>) distributions of the $Ti_3C_2T_x$ MXene flakes, resulting log-normal distributions peaked at 2.61 nm and 2.81 μm for <*t*> and <*S*>, respectively. The results indicate that the as-prepared $Ti_3C_2T_x$ MXene solutions consists of ~70% monolayer and bilayer flakes, considering a monolayer thickness of 1.6 nm [1] and an additional 1 nm thickness attributed to water molecules trapped between the flake and the substrate [2].

Figure S1(b) presents a high-resolution AFM micrograph of a monolayer $Ti_3C_2T_x$ flake with a <*t*> of 2.43 nm and a <*S*> of 2.9 μm, as shown in the height profile in Figure S1(c).

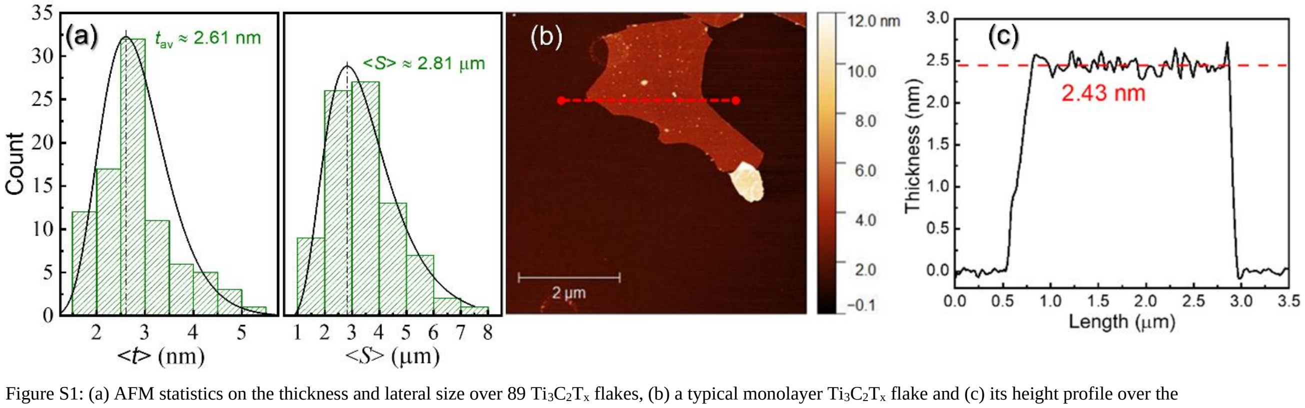

Figure S1: (a) AFM statistics on the thickness and lateral size over 89 $Ti_3C_2T_x$ flakes, (b) a typical monolayer $Ti_3C_2T_x$ flake and (c) its height profile over the lateral length.

Figure S2(a) report the XPS survey spectra for both Ti3C2Tx-NSs 1:5 blend and NSs; Figure S2(b) the Zn 2p and (c) the O1s region for NSs.

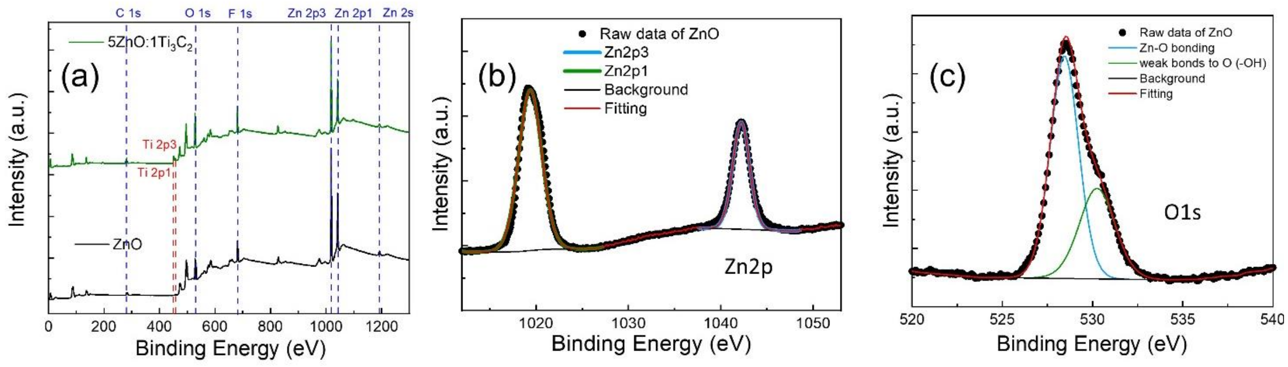

Figure S2: (a) $Ti_3C_2T_x$-NSs 1:5 blend and NSs XPS survey spectra; (b) the Zn 2p and (c) the O1s region for NSs.

Figure S3 shows the CV curves acquired for all $Ti_3C_2T_x$-NSs blends, NSs and $Ti_3C_2T_x$ in 1M Na_2SO_4 at different scan rates, from 5 to 100 mV s^{-1}.

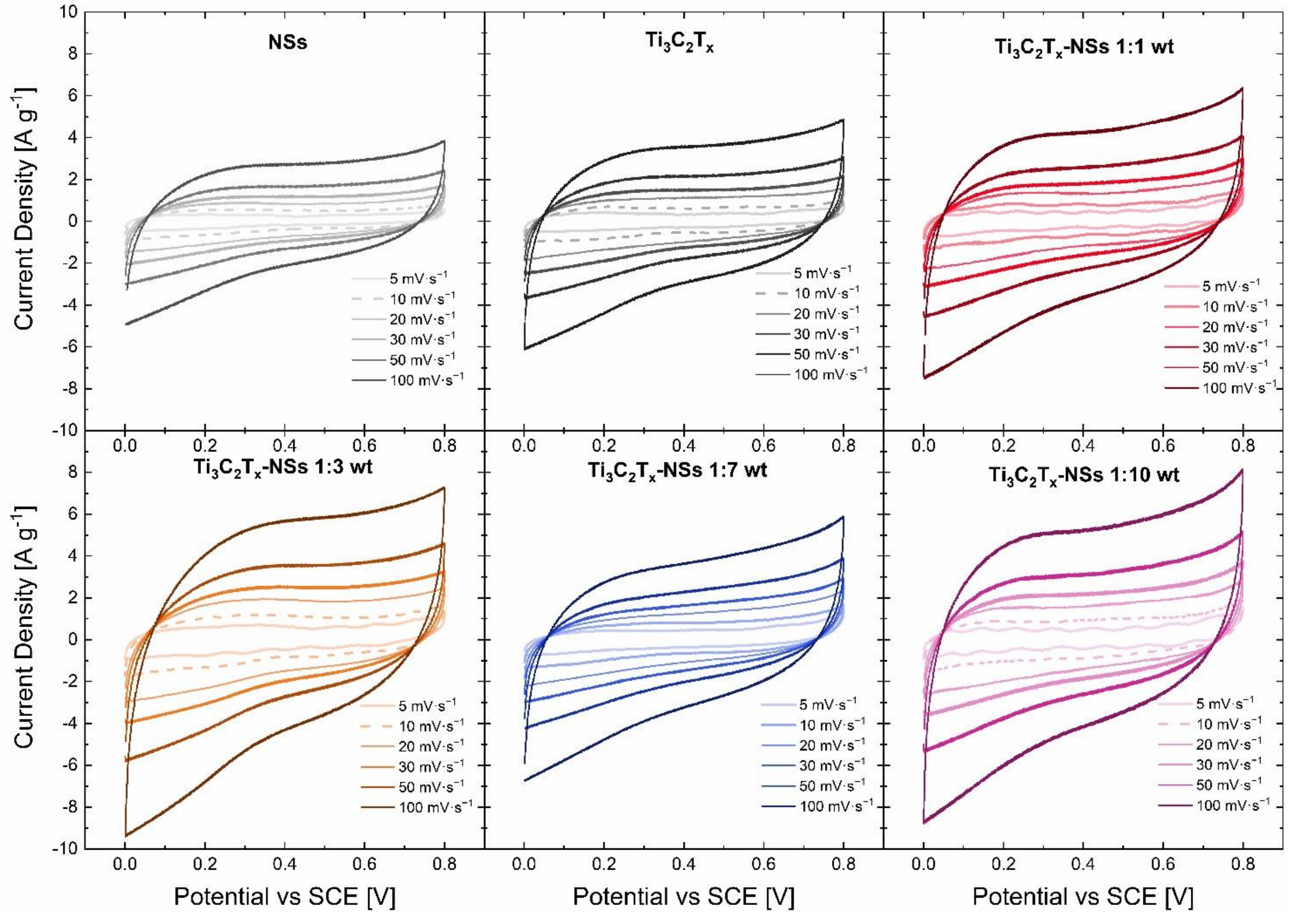

Figure S3: CV curves acquired for all $Ti_3C_2T_x$-NSs blends, NSs and $Ti_3C_2T_x$ in 1M Na_2SO_4 at different scan rates, from 5 to 100 mV s^{-1}.

Figure S4(a-c) report the GCD curves for the best performing blend ($Ti_3C_2T_x$-NSs 1:5), and bare $Ti_3C_2T_x$ and NSs as references. All curves explored the same potential windows used for the CV analyses.

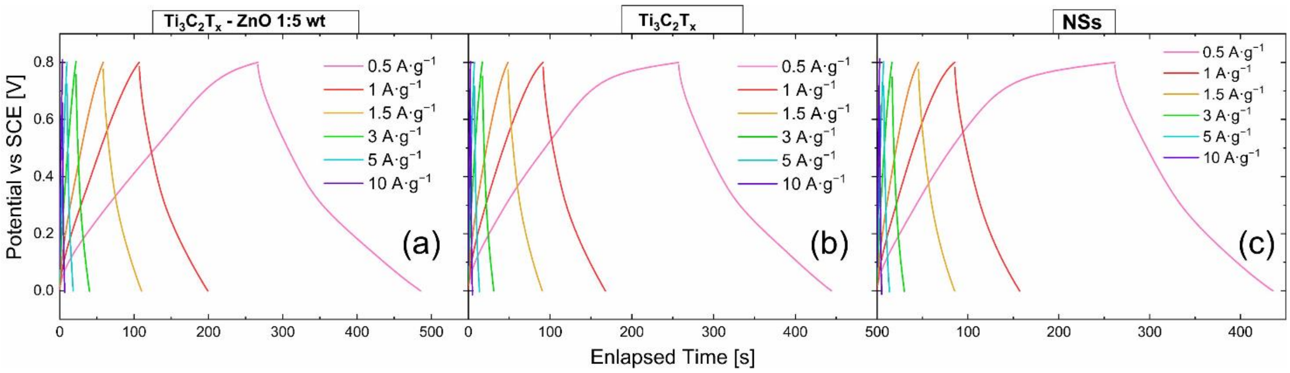

Figure S4: GCD curves of (a) $Ti_3C_2T_x$-NSs 1:5, (b) $Ti_3C_2T_x$, and (c) NSs, at 0.5 (pink), 1 (red), 1.5 (orange), 3 (green), 5 (light blue) and 10 $A \cdot g^{-1}$ (violet) current densities.

References

[1] A. Lipatov, M. Alhabeb, M. R. Lukatskaya, A. Boson, Y. Gogotsi, A. Sinitskii, *Adv. Electron. Mater.* **2016**, *2*.

[2] T. Carey, S. Cacovich, G. Divitini, J. Ren, A. Mansouri, J. M. Kim, C. Wang, C. Ducati, R. Sordan, F. Torrisi, *Nature communications*, **2017**, *8*.
